# Public Interest in Acetyl Hexapeptide-8: Longitudinal Analysis

**DOI:** 10.2196/54217

**Published:** 2024-02-20

**Authors:** Sofia Eva Olsson, Bhavana Sreepad, Trevor Lee, Manal Fasih, Arman Fijany

**Affiliations:** 1 Anne Burnett Marion School of Medicine Fort Worth, TX United States

**Keywords:** acetyl-hexapeptide-8, anti-aging, anti-wrinkle, Argireline, BoNT, botox, botulinum neurotoxin, cosmetic dermatology, cosmetic, dermatologist, dermatology, injectable neurotoxin, neurotoxin, skin specialist, topical agent, topical

## Abstract

**Background:**

Acetyl hexapeptide-8, also known as Argireline, is a topical, short-acting, synthetic peptide that has recently gained popularity for its antiwrinkle effects. This agent has emerged as a more accessible alternative to botulinum neurotoxin.

**Objective:**

This study evaluates the public interest in acetyl hexapeptide-8 in the United States from 2013 to 2023, as described by search volume on Google, the most-used search engine.

**Methods:**

We analyzed the longitudinal relative monthly search volume from January 1, 2013, to January 1, 2023, for acetyl hexapeptide–related terms. We compared the internet search trends for “Botox” during this period to “Argireline.”

**Results:**

The terms “Argireline” and “Botox in a Bottle” both had substantial increases in search volume in 2022. Although its search volume is drastically increasing, “Argireline” was less searched than “Botox,” which had a stable, up-trending search volume over the past decade.

**Conclusions:**

The increasing interest in acetyl hexapeptide-8 may be due to its cost-effectiveness and use as a botulinum neurotoxin alternative. Affordability, over-the-counter availability, and ease of self-application of the agent suggest its potential to enhance accessibility to cosmetic dermatologic care.

## Introduction

Botulinum neurotoxins (BoNTs) have long been considered the most effective cosmetic intervention to reduce wrinkles and fine lines [1]. However, many individuals face barriers such as cost and transportation when seeking BoNT treatment.

Acetyl hexapeptide-8, which acts similarly to BoNTs, has gained traction due to its low cost, topical application method, and increased safety of use [2]. The peptide may be referred to as acetyl hexapeptide-3 or acetyl hexapeptide-8 amide, and it is more commonly identified by its trade name, Argireline, produced by the Lubrizol Corporation. The topical peptide is a synthetic compound mimicking the N-terminus of synaptosomal-associated protein of 25 kDa (SNAP-25) [3]. This structure allows for inhibition of the soluble N-ethylmaleimide-sensitive factor activating protein receptor (SNARE) ternary complex assembly and consequently inhibits Ca^2+^-dependent exocytosis of acetylcholine into the neuromuscular junction [2,3]. This mechanism is similar to that of BoNT type A, yielding comparable outcomes that are shorter-acting with milder neurotoxicity [2,3]. As of 2020, acetyl hexapeptide-8 was reported as an ingredient in 452 cosmetic products [4]. Though there are limited data on the price ranges of these products, a recently popularized brand of 10% Argireline water-based serum costs US $9.40 for an approximately 4-month supply. Prices may vary, but acetyl hexapeptide-8 products appear to cost less than cosmetic BoNT injections, which range from US $300 to US $600 per treatment [5]. This affordability expands access to antiwrinkle care across a broader socioeconomic demographic. Additionally, the product is considered safe for topical use with minimal risk of complications or adverse effects [4,6,7].

A large-scale study published in 2013 revealed the efficacy of acetyl hexapeptide-8 in reducing periorbital wrinkles [8]. However, Argireline became popular on TikTok, a social media platform where users share short clips, in 2022 [9,10]. The term “Botox in a Bottle” was coined to describe the product on TikTok, where users praised the compound for its antiaging properties by reducing wrinkles and fine lines [11]. Acetyl hexapeptide-8 is marketed as a low-cost alternative to BoNT treatments for those hesitant or unable to afford injection therapies [11].

With casual reporting of increased acetyl hexapeptide-8 popularity [11], it is imperative to quantitatively analyze trends in public interest in the agent. Such analysis serves as a reflection of trends in consumer interest and use [12]. With Google being the most widely used search engine globally and in the United States [13], it serves as a primary platform for individuals interested in acetyl hexapeptide-8 products to seek further information. This study is the first to comprehensively examine public interest in acetyl hexapeptide-8 on the internet, offering a realistic view of its trends in the United States and the necessity for further medical research on the product.

## Methods

The relative monthly volume of acetyl hexapeptide-related Google searches was determined using the Google Trends database [14]. Google Trends is a tool that provides insight into longitudinal search volume data on Google and has been used in recent literature to study human behaviors and interests without consumer barriers such as cost and transportation [14-16].

In this analysis, search volume data were collected between January 1, 2013, and January 1, 2023. The following search terms were examined: “Argireline,” “Botox in a Bottle,” “Acetyl hexapeptide-3,” and “Acetyl hexapeptide-8.” These terms were selected to encompass the scientific nomenclature, trade name, and colloquial phrases relating to acetyl hexapeptide-8. Additionally, the term “Botox” was included to provide a basis for comparison between traditional BoNT injections and the newer topical alternative, Argireline.

Monthly search volumes for each of these terms were obtained from Google Trends as normalized values on a relative search index. The index scale used for analysis ranged from 0, representing minimal search volume, to 100, indicating maximal search volume.

## Results

**Figure 1:**
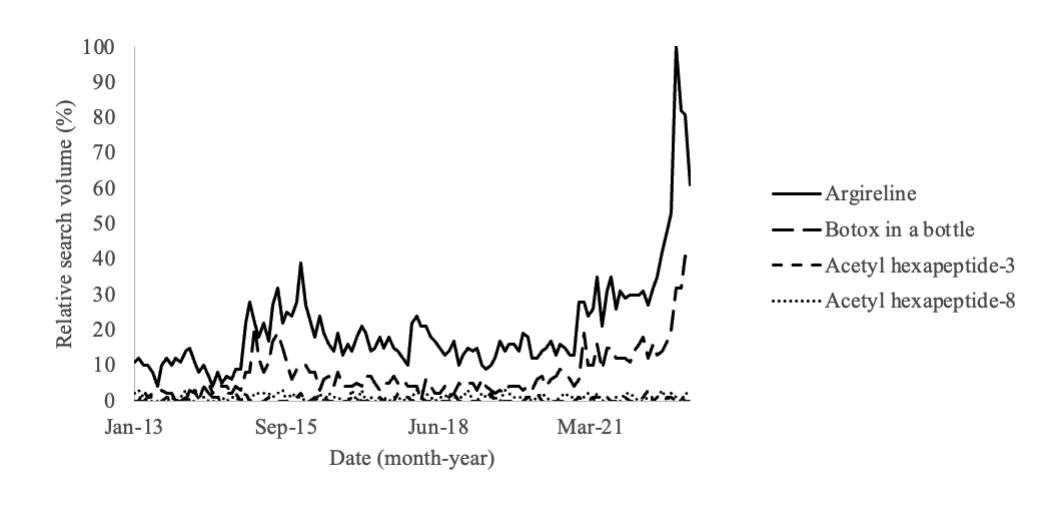
Relative search volume of acetyl hexapeptide–related terms on Google from January 1, 2013, to January 1, 2023.

Search terms “Argireline” and “Botox in a Bottle” followed similar trends in web-based popularity, while “Acetyl hexapeptide-8” and “Acetyl hexapeptide-3” did not (Figure 1). There appeared to be relatively sparse online interest in acetyl hexapeptide-related search terms before February 2015. Following this spike, public interest, as described by search volume, stabilized before rising in May 2021, with a peak in October 2022. Google users primarily searched for acetyl hexapeptide-8 by its trade name, “Argireline,” followed by the colloquial name, “Botox in a Bottle.” The terms “Acetyl hexapeptide-3” and “Acetyl hexapeptide-8” had the lowest search volumes with relatively stable searches over the past decade.

**Figure 2:**
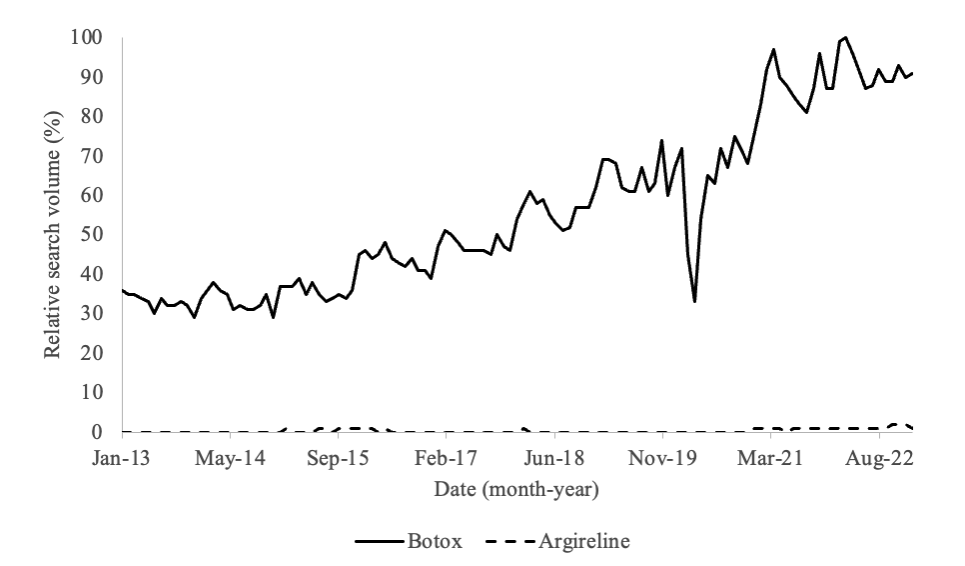
Comparison of relative Google search volume for search terms “Argireline” and “Botox” from January 1, 2013, to January 1, 2023.

Acetyl hexapeptide-8 is frequently compared to BoNTs due to their similar mechanism of action and overlapping use as antiwrinkle agents. However, despite its recent uptrend in Google searches (Figure 1), “Argireline” is searched less than the term “Botox,” which has steadily up-trended over the past decade (Figure 2). Botox appears to have relatively substantial and consistent internet popularity compared to the newly popularized Argireline peptide.

## Discussion

### Overview

This study is the first to describe the longitudinal internet popularity of the topical antiwrinkle agent acetyl hexapeptide-8 over the past decade. Viewers likely searched the internet to purchase or research Argireline peptide following exposure through social media or other sources. Though important studies demonstrating the antiwrinkle effects of acetyl hexapeptide-8 were published in 2013, the search volume of the product’s trade name, Argireline, increased exponentially in the year 2022 (Figure 1) [7]. This was likely due to the popularization of the serum through social media platforms such as TikTok. The longitudinal increase in Argireline and related terms’ search volumes confirms a growing public interest in the agent, likely as an alternative to traditional BoNTs. Despite its marketing as a cost-effective, less-invasive, and shorter-acting alternative to BoNTs [1,11], Google Trends data analysis revealed that the internet popularity of “Botox” increased within the last decade as well. Botox was searched for far more frequently than the newly popularized Argireline. This may be due to the perceived reliability of BoNTs, as they have been approved by the Food and Drug Administration for cosmetic use since 2002 [17,18].

The relatively low search volume for the terms “Acetyl hexapeptide-3” and “Acetyl hexapeptide-8” may stem from the knowledge barrier of scientific jargon and specialized terminology [19,20]. Products containing acetyl hexapeptide-8 appear to use the agent’s scientific nomenclature or its trade name in ingredient lists, with no consensus on the use of a single term. Internet users may be familiar with terms or phrases commonly used in English, such as “Botox in a Bottle” or “Argireline,” and rely on them to better comprehend the effects of the product [19,20]. Importantly, the conflicting public search trends between lay and scientific jargon may indicate a need for further scientific research on the agent and clarification to consumers regarding their acetyl hexapeptide-8 product options.

The less-invasive nature of acetyl hexapeptide-8, the ability to self-apply cost-effectively, and the minimal side effects are potential reasons for its increasing popularity over the past decade. Due to its lesser neurotoxicity and shorter-acting effects, acetyl hexapeptide-8 does not carry the risks of ptosis, eyebrow asymmetry, and other complications seen in facial BoNT injections [4,6,21]. The ability to self-apply acetyl hexapeptide-8 products brings down the cost of their usage, as sterile equipment and a medical professional are not required for their application. Argireline peptide solutions typically cost less than US $100 when purchased over the counter, whereas BoNT injections require a medical professional for administration, costing an average of US $300-US $600 [5,22]. The relatively low price point and over-the-counter status of acetyl hexapeptide-8 products allow them to improve accessibility to cosmetic dermatologic care. Self-application also improves accessibility to antiwrinkle care, as transportation to a site and appointment time are no longer barriers to treatment.

There are various strengths to this project. The anonymity of Google Trends big data limits interviewer and chronology bias. Observing internet search volume gauges consumer interest and exposure without the financial barrier of product purchase. As of 2022, Google is the most-used search engine, occupying 86.99% of the United States search engine market [13]. Therefore, Google search volumes provide the most complete understanding of public interest and internet exposure to acetyl hexapeptide-8. A limitation of Google Trends’ big data is the lack of community and individual-level data, hindering assessment groups with differing representation. It also allows for potential bias from differences in the interests of Google users compared to those who use other search engines.

Understanding consumers’ skincare preferences can guide future research regarding trending products’ efficacy, safety, and innovation. Future directions for acetyl hexapeptide-8 research include its potential use as a therapeutic agent alongside the current cosmetic indications. Assessing Argireline use in various socioeconomic groups, age groups, and geographic locations may provide greater insight into its role as an accessible option for dermatologic health maintenance.

### Conclusion

This study was the first to analyze public interest in acetyl hexapeptide-8, as described by the relative search volume of acetyl hexapeptide-related terms on Google over the past decade. Though the agent’s antiwrinkle effects were published in 2013, results indicate a recent surge in internet popularity in 2022. Acetyl hexapeptide-8 can improve access to antiwrinkle care due to its low price point, over-the-counter status, and ability to be self-applied. The authors recommend additional research assessing the safety profiles of acetyl hexapeptide-8 products as well as their use and interest among various demographics.

## Abbreviations

BoNT: botulinum neurotoxin
SNAP-25: synaptosomal-associated proteins of 25 kDa
SNARE: soluble N-ethylmaleimide-sensitive factor activating protein receptor
